# Transcriptome analysis of *Sparidentex hasta* larvae exposed to water-accommodated fraction of Kuwait crude oil

**DOI:** 10.1038/s41598-024-53408-2

**Published:** 2024-02-13

**Authors:** Vinod Kumar, Qusaie Karam, Anisha B. Shajan, Sabeeka Al-Nuaimi, Zainab Sattari, Saleem El-Dakour

**Affiliations:** Environment and Life Sciences Research Center, Kuwait Institute for Scientific Research, P.O. Box 24885, 13109 Safat, Kuwait

**Keywords:** Molecular biology, Transcriptomics, Environmental sciences, Environmental impact

## Abstract

Anthropogenic activities have been shown to significantly affect marine life. Water pollution and oil spills are particularly deleterious to the fish population, especially during their larval stage. In this study, Sobaity-sea bream *Sparidentex hasta* (Valenciennes, 1830) larvae were exposed to serial dilutions of water-accommodated fraction of Kuwait crude oil (KCO-WAF) for varying durations (3, 6, 24, 48, 72 or 96 h) in acute exposure regime. Gene expression was assessed using RNA sequencing and validated through RT-qPCR. The RNA sequencing data were aligned to the sequenced genome, and differentially expressed genes were identified in response to treatment with or without KCO-WAF at various exposure times. The highest number of differentially expressed genes was observed at the early time point of 6 h of post-exposure to KCO-WAF. The lowest number of differentially expressed genes were noticed at 96 h of treatment indicating early response of the larvae to KCO-WAF contaminant. The acquired information on the differentially expressed genes was then used for functional and pathway analysis. More than 90% of the differentially expressed genes had a significant BLAST match, with the two most common matching species being *Acanthopagrus latus* and *Sparus aurata*. Approximately 65% of the differentially expressed genes had Gene Ontology annotations, whereas > 35% of the genes had KEGG pathway annotations. The differentially expressed genes were found to be enriched for various signaling pathways (e.g., MAPK, cAMP, PI3K-Akt) and nervous system-related pathways (e.g., neurodegeneration, axon guidance, glutamatergic synapse, GABAergic synapse). Early exposure modulated the signaling pathways, while KCO-WAF exposure of larvae for a longer duration affected the neurodegenerative/nervous system-related pathways. RT-qPCR analysis confirmed the differential expression of genes at each time point. These findings provide insights into the underlying molecular mechanisms of the deleterious effects of acute exposure to oil pollution—on marine fish populations, particularly at the early larval stage of *Sparidentex hasta*.

## Introduction

Anthropogenic activities are widely acknowledged to have a significant adverse impact on marine ecosystems and biodiversity globally^1,2^. Water pollution from various sources, such as agricultural, municipal, and industrial sources has become a major concern, which affects marine life. Further, the effects are profoundly evident in fish populations^3^. Multiple studies have reported a decline in the marine fauna as a result of anthropogenic activities owing to urbanization, and industrialization^4,5^.

Crude oil is one of the major pollutants released into the water and drastically affects the marine ecosystem. The Deepwater Horizon oil spill, also known as the largest oil spill in history, occurred in 2010 in the Gulf of Mexico. Approximately 4.4 million barrels of oil were released into the sea, resulting in a catastrophic impact on the marine ecosystem and coastal communities^6^. Despite the passing of a decade since this catastrophic event, researchers are still uncovering a vast amount of information regarding its effect on the marine environment^6,7^. For example, in the aftermath of the Deepwater Horizon disaster, it was discovered that the offshore fish population was in a lymphopenic or immunocompromised state due to the exposure to polycyclic aromatic hydrocarbons (PAHs)^8^. A study conducted on the short-term impact of the 2019 oil spill in Brazil on fisheries, indicated the prevalence of low molecular weight PAHs, mainly naphthalenes in the edible tissues of 34 finfish and shellfish species within the first 3 months time^9^. Oil spills not only affect the fish species but also other marine fauna. Oil spills are responsible for the mortalities of four different species of sea snakes in the Gulf of Oman^10^. The study showed that the majority (~ 85%) of sea snakes had oil covering on 75–100% of their bodies. Furthermore, snouts and eyes of ~ 91.4% of sea snakes were observed with oil covering. In addition, a large proportion (26–41%) of sea snakes had oil content in their mouth, esophagus and stomach. Furthermore, climate change and crude oil spills have a combined effect on fish species^11^. Polar cod embryos and larvae were exposed to low crude oil levels and a 2.3 °C increase in water temperature. The synergistic effects of increased temperature and crude oil exposure on early life stages were most prevalent in the first feeding larval stages, highlighting potential long-term consequences for survival, growth, and reproduction^11^. In addition, produced water in oil drilling, contains high levels of aromatic hydrocarbon and heavy metals. Its release can particularly impact the vulnerable larval and juvenile stages of marine organisms, leading to long-term ecological consequences^12^. These findings highlight the urgent need for effective measures to prevent oil spills and mitigate their long-lasting effects on marine fauna.

Kuwait is located in the North of the Arabian Gulf with an extensive coastline of 290 km long, taking into account the inclusion of its associated islands^13^. The effects of anthropogenic activities on marine life, particularly fish population has also been investigated in Kuwaiti waters. Alqattan and Gray discussed the nature of pollution in Kuwaiti waters, its causes, and measures that could be taken to reduce it^14^. The article also investigates whether pollution is the primary reason for the decline in fish stocks in Kuwaiti waters. Although the study indicates the existence of pollution in Kuwaiti waters, it fails to establish a direct link between oil pollution and the decline in fish stocks. In another study, Edmonds et al. assessed the state of Kuwait's marine biodiversity by reviewing data on the occurrence, distribution, and threats to key marine habitats and associated indicator organisms^15^.

The effects of oil spills on increased mortality of fish has been demonstrated in few other studies too^16,17^. However, eggs and larvae of fish species exhibited more vulnerability to the toxicity of crude oil due to their smaller size, poorly developed membrane and detoxification mechanisms^17^. Multiple studies have shown that PAHs in the crude oil even at low concentrations can cause lethal or sub-lethal damage to fish eggs and larvae^18–21^. This could result in morphological deformities, reduced feeding and growth rates resulting in starvation, and further increases the vulnerability to predators. A few studies have also indicated increased mortality of eggs and larvae at the spill sites^22,23^.

High-throughput technologies, such as microarray and RNA sequencing are powerful tools to understand the effect of oil pollution on the biology of fish species. RNA sequencing was performed to study the effect of water-accommodated fraction (WAF) of crude oil exposure in the gills of Japanese flounder, *Paralichthys olivaceus*, and the study provided insights into the mechanisms of WAF-induced toxicity in *P. olivaceus*^24^. Crude oil exposure weakens the immune function and increases the susceptibility to *Vibrio anguillarum*, a causative agent of vibriosis in southern flounder^25^. The authors demonstrated decreased expression of immunoglobulin M, the major systemic fish antibody, and downregulation of the genes related to immune function, response to stimulus and hemostasis in the fish specimens exposed the crude oil. RNA sequencing of liver and gill tissues of lined sole fish was performed to assess the transcriptomic changes in response to WAF of light crude oil^26^. WAF treatment resulted in the induction of hypoxia-regulated genes and the regulation of multiple signaling pathways in lined sole fish. Jantzen et al.,^27^ demonstrated the effect of three different polyfluorinated compounds on morphometric, behavior, and gene expression in both yolk sac fry and larval zebrafish. Administration of crude oil to Atlantic haddock in short term exposures at two developmental stages resulted in the impairment of calcium homeostasis by affecting calcium-regulated developmental pathways, including cardiogenesis^2^.

The Sobaity seabream, *Sparidentex hasta* (Valenciennes, 1830), is an economically important species and is considered as one of the most promising species for aquaculture due to its good adaptation to captivity, rapid growth, and high market value. The toxicity of WAF and chemically enhanced water-accommodated fraction (CEWAF) of Kuwait crude oil with three dispersants (Corexit^®^ 9500, Corexit^®^ 9527, and Slickgone^®^ NS) on the larvae of *S. hasta* was investigated^28^. The authors demonstrated that the dispersed and undispersed KCO resulted in toxicity manifestations in different magnitudes. WAF of the Kuwait crude oil and CEWAF with of Corexit^®^ 9527 had higher toxicity, whereas, CEWAFs with Corexit^®^ 9500 and Slickgone^®^ NS had lower toxicity on fish larvae^28^. Nevertheless, there remains a research gap in investigating the effects of crude oil on *S. hasta* larvae at the molecular-level.

In the current study, we used RNA sequencing to investigate the effects of WAF of Kuwait crude oil (KCO-WAF) on *S. hasta* larvae at various exposure times. The larvae were incubated in seawater with or without KCO-WAF for 3, 6, 24, 48, 72 or 96 h and differentially expressed genes were identified using RNA sequencing. The functional and pathway analyses of the genes were performed to determine the effects of KCO-WAF on fish larvae at the molecular level. Additionally, we analyzed the effects of early, intermediate, and longer exposures of KCO-WAF by studying the various sets of differentially expressed genes. Furthermore, we have also validated a selected set of genes differentially expressed at each time point through RT-qPCR.

## Materials and methods

### Fish larvae exposure assay

Wild broodstock fish caught from the Kuwait waters were domesticated in tanks for 2 years at the Mariculture and Fisheries Department, Kuwait Institute for Scientific Research (KISR), Salmiya, Kuwait. All experiments on fish and larvae were performed as per the approved institutional guidelines of KISR. The cultured broodstock spawned naturally in tanks without any hormonal induction. Twenty-four hours post-hatch larvae of Sobaity-sea bream weighing around 0.1–0.75 mg were procured from the Aquaculture facility of KISR and acclimated to the laboratory conditions at the Ecotoxicology Laboratory at KISR. Kuwait Crude Oil (KCO) of API gravity 30 was procured from Petroleum Research Center (PRC) of KISR. The acute toxicity test was done according to the guidelines of the OCED (Organization of Economic Co-Operation and Development) for the Fish Embryo Toxicity (FET) Test^29,30^. KCO WAF prepared according to Singer et al.^31^, and previously standardized method within the research team's laboratory^32,33^. A known amount of KCO (0.25, 0.5, or 1 g) was placed in aspirator bottles containing 1 L filtered seawater, stirred for 24 h and then allowed to stay for the separation of the aqueous layer. WAF fraction was drained and collected in amber bottles, characterized for TPH and used in WAF exposure experiments. Based on the results of the preliminary experiments, the WAF prepared by mixing 0.5 g KCO/L concentration was used for the assay at 50% dilution of WAF (1:1) with clean sea water containing larvae (for each replicate *n* = 40–50). The larvae were then exposed to KCO-WAF in triplicate for 3, 6, 24, 48, 72, or 96 h in a static non-renewal test regime according to ASTM: E 729-96^34^. The control treatment consisted of larvae samples exposed to filtered seawater without KCO-WAF for the same duration at each time point. Each treatment was conducted in individual 100 ml beakers containing 40–50 larvae in filtered sea water with our without WAF.

### RNA isolation

The total RNA was isolated from the control and KCO-WAF-exposed larvae using TRIzol LS reagent (Invitrogen, USA). Each experimental replicate beaker containing 40–50 larvae was sieved for collecting the larvae and immediately frozen in liquid nitrogen. The samples were homogenized with 0.75 mL TRIzol LS reagent and stored at − 80 ℃ until further use. RNA isolation was carried out following the instructions provided by the manufacturer. The total yield of RNA was determined using a Nanodrop spectrophotometer and Qubit fluorometer, and the purity of RNA was assessed by measuring the ratio of absorbance at 260 nm and 280 nm and by agarose gel electrophoresis.

### Library preparation and sequencing

The mRNA molecules were purified from total RNA using oligo(dT)-attached magnetic beads and fragmented using fragmentation reagent. First-strand cDNA was generated using random hexamer-primed reverse transcription, followed by a second-strand cDNA synthesis. The synthesized cDNA was subjected to end-repair and was 3′ adenylated. Adapters were ligated to the ends of the 3′ adenylated cDNA fragments followed by amplification. The PCR products were purified with Ampure XP Beads (AGENCOURT), and dissolved in EB solution. Library was validated on the Agilent Technologies 2100 bioanalyzer. The double-stranded PCR products were heat denatured and circularized by the splint oligo sequence. The single-strand circular DNA (ssCir DNA) molecules were considered as the final library. The library was then amplified with phi29 to make DNA nanoball (DNB), which had more than 300 copies of one molecule. The DNBs were loaded into the patterned nanoarray paired-end reads 100/150 bp were generated. The sequencing was carried out at the Beijing Genomics Institute (BGI) Tech Solutions, Hong Kong. The sequencing data can be accessed from NCBI-SRA under the accession PRJNA748027.

### Analysis of transcriptome sequencing data and differential gene expression

Around 200 Gb of paired-end RNA sequencing data was checked for quality before and after trimming using FastQC v0.10.1 [https://www.bioinformatics.babraham.ac.uk/projects/fastqc/]. The RNA-seq raw data was trimmed for low quality reads based on base-quality scores and read length, using Trimgalore 0.6 (https://www.bioinformatics.babraham.ac.uk/projects/trim_galore/). A minimum quality score of 20 and read length of 50 was considered for trimming. The trimmed and filtered RNA-seq reads were aligned to the assembled genome^35^ and transcriptome using TopHat version 2.1.0^36^ with default parameters. Quantification of the genes was performed using Cufflinks version 2.2.1^37^ with default parameters. The differential expression analysis of the genes was performed using Cuffdiff package within Cufflinks^37^, with default parameters. Genes differentially expressed with a |log2-fold ≥ 1| and FDR *P* value < 0.01 were considered significant in each comparison.

### Functional analysis of differentially expressed genes

The significantly differentially expressed genes were considered for functional analysis, performed using Blast2GO tool^38^. The sequences corresponding to significant genes were mapped to ‘nr’ protein database using blastx with an *E-value* of 1.0E-5, word size of 6 and HSP length cut-off of 33. The gene sequences were blasted against “Metazoa (tax id: 33208)” sequences in the ‘nr’ database. The blast alignment results were then used for Gene Ontology (GO) mapping (GO database version 2021-11) and annotation. Further, InterproScan analysis was performed using the alignment results.

### Reverse transcription-quantitative polymerase chain reaction (RT-qPCR)

For experimental validation of the RNA-seq data, 12 differentially expressed genes were selected for RT-qPCR experiments. Genes for RT-PCR were selected based on their high expression levels determined through RNA sequencing data. Beta-actin was considered as one of the reference gene. We selected another control gene from the list of un-differential genes showing the highest *p* value and lowest logFC across all treatment conditions. Primers for the genes were designed using the Ex-Ex Primer tool^39^. A list of primers used is provided in Supplementary file 1. RT-qPCR was performed using TB Green Premix Ex Taq II (Tli RNase H Plus) in a StepOnePlus RealTime PCR System (Applied Biosystems). The thermal cycling conditions were set as follows: sample holding at 95 °C for 10 min; 40 cycles of denaturation step at 95 °C for 20 s; annealing at 63 °C for 20 s; extension at 72 °C for 20 s. The Ct value was calculated with auto threshold using the default option. The calculation of the fold change was performed as previously described^40^. The fold change of the genes was calculated using the 2^(-DeltaDeltaC(T))/comparative C(T) method. For both control and KCO-WAF exposed samples, the expression level was calculated using the following formula: “2^-(Ct (gene of interest − reference gene)”, and the fold change for each gene was derived by dividing its expression in the KCO-WAF exposed samples with that of in control samples.

## Results

### Transcriptome sequencing of KCO-WAF treated and untreated *S. hasta* larval samples

**Figure 1:**
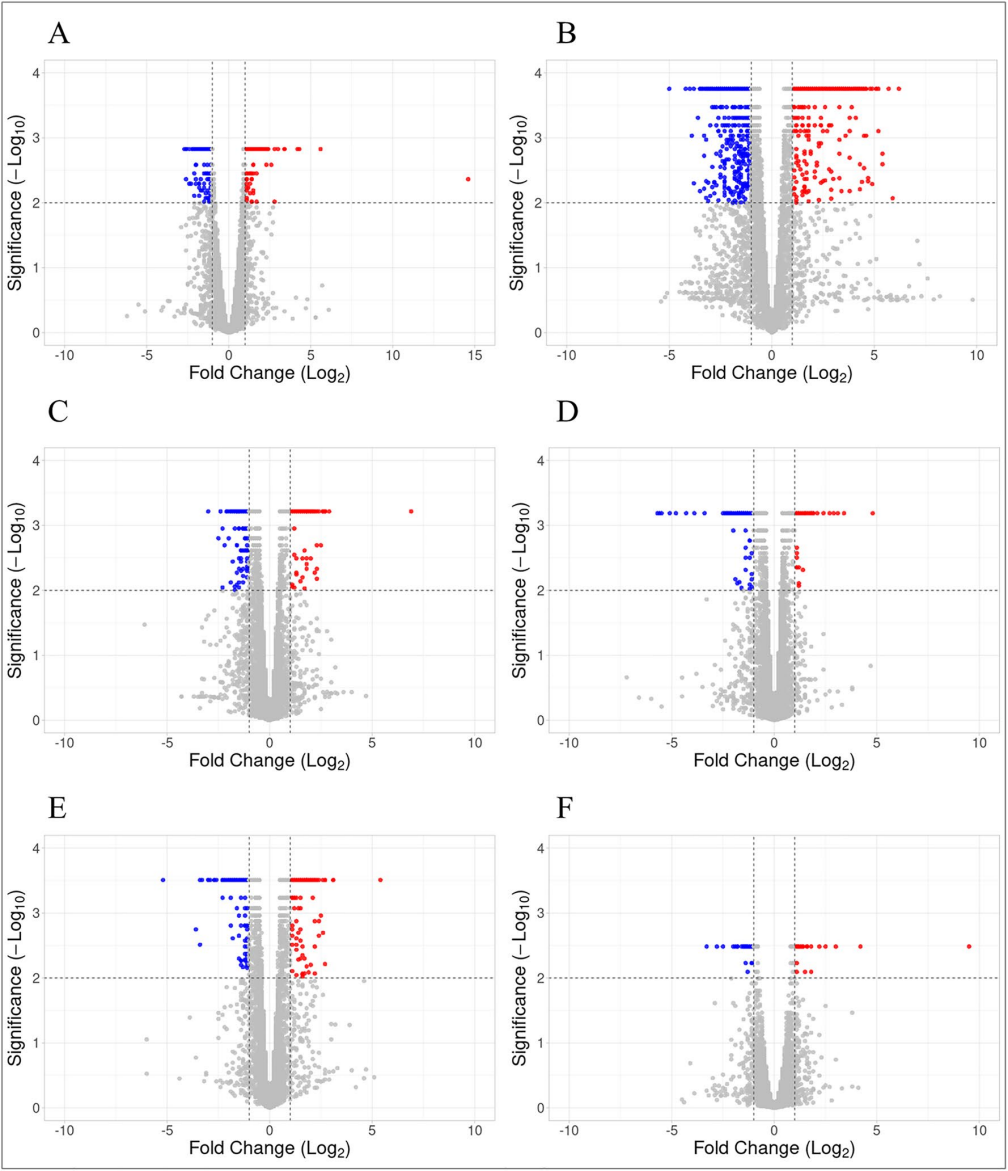
Volcano plots showing significantly differentially expressed genes of *S. hasta* larvae exposed to KCO-WAF and control treatment. The x-axis represents log2-fold change, whereas y-axis shows -log10 FDR *P* value. A: 3 h; B: 6 h; C: 24 h; D: 48 h; E: 72 h; F: 96 h.

A total of 35 samples across six treatment and control conditions were sequenced to obtain approximately two billion paired-end reads. On average, 60 million raw reads were obtained per sample, and ~ 58 million good-quality reads per sample were retained after quality filtering. Except for 24 h KCO-WAF treated sample, all samples were sequenced in triplicate. The quality filtering of the reads resulted in approximately 96.3% of good-quality reads (Table 1). The number of the raw and filtered reads obtained for each replicate along with the read length are listed in Supplementary File 2. The filtered reads were aligned to an assembled genome^35^ containing 41201 genes. Differential expression analysis between treatment and control conditions resulted in varying numbers of genes across different treatment groups (Fig. 1). There was no specific trend in the number of genes expressed differently across the increasing treatment durations. The 96 h treatment resulted in least number of differentially expressed genes, whereas 6 h KCO-WAF treatment resulted in most differential genes (Table 2 and Supplementary File 3). Overall, the number of downregulated genes was more than that of the upregulated genes across the treatment groups.

**Table 1:** Summary of raw and filtered RNA sequencing data.

Group name	No. of raw reads	No. of filtered reads	% of retained reads
SH3C	126,403,462	123,481,984	97.69
SH3T	123,085,920	118,377,054	96.17
SH6C	123,798,064	119,916,500	96.86
SH6T	122,898,860	117,875,658	95.91
SH24C	293,467,124	284,506,846	96.95
SH24T	198,314,268	190,768,940	96.20
SH48C	314,612,888	303,529,324	96.48
SH48T	330,566,174	317,265,238	95.98
SH72C	124,409,630	121,329,818	97.52
SH72T	122,567,472	117,015,820	95.47
SH96C	123,623,556	118,302,348	95.70
SH96T	122,356,234	116,080,006	94.87

*SH* Sparidentex hasta, *C* Control, *T* Exposed to KCO-WAF.

**Table 2:** Differentially expressed genes (log FC ≥ 1; FDR *P* < 0.01) in *S. hasta* larvae exposed to KCO-WAF and control treatment.

Duration of the exposure (h)	Differentially expressed genes	
	Upregulated	Downregulated
3	121	127
6	731	759
24	124	166
48	67	151
72	240	271
96	34	36

### Functional analysis of significantly differentially expressed genes

**Figure 2:**
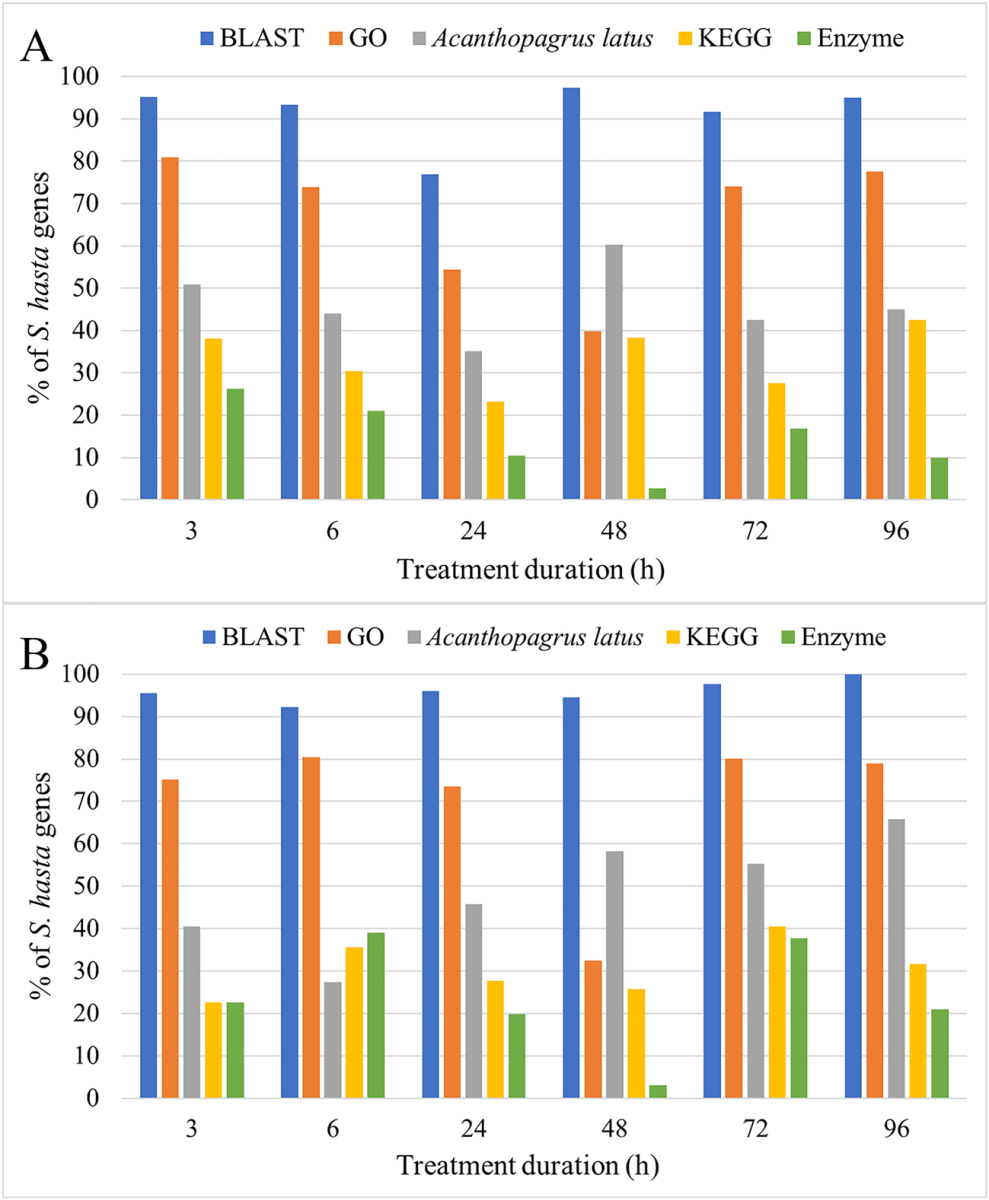
Mapping and annotation of significantly differentially expressed genes of *S. hasta* at different treatment durations. (**A**) Upregulated. (**B**) Downregulated. GO: Gene Ontology.

**Figure 3:**
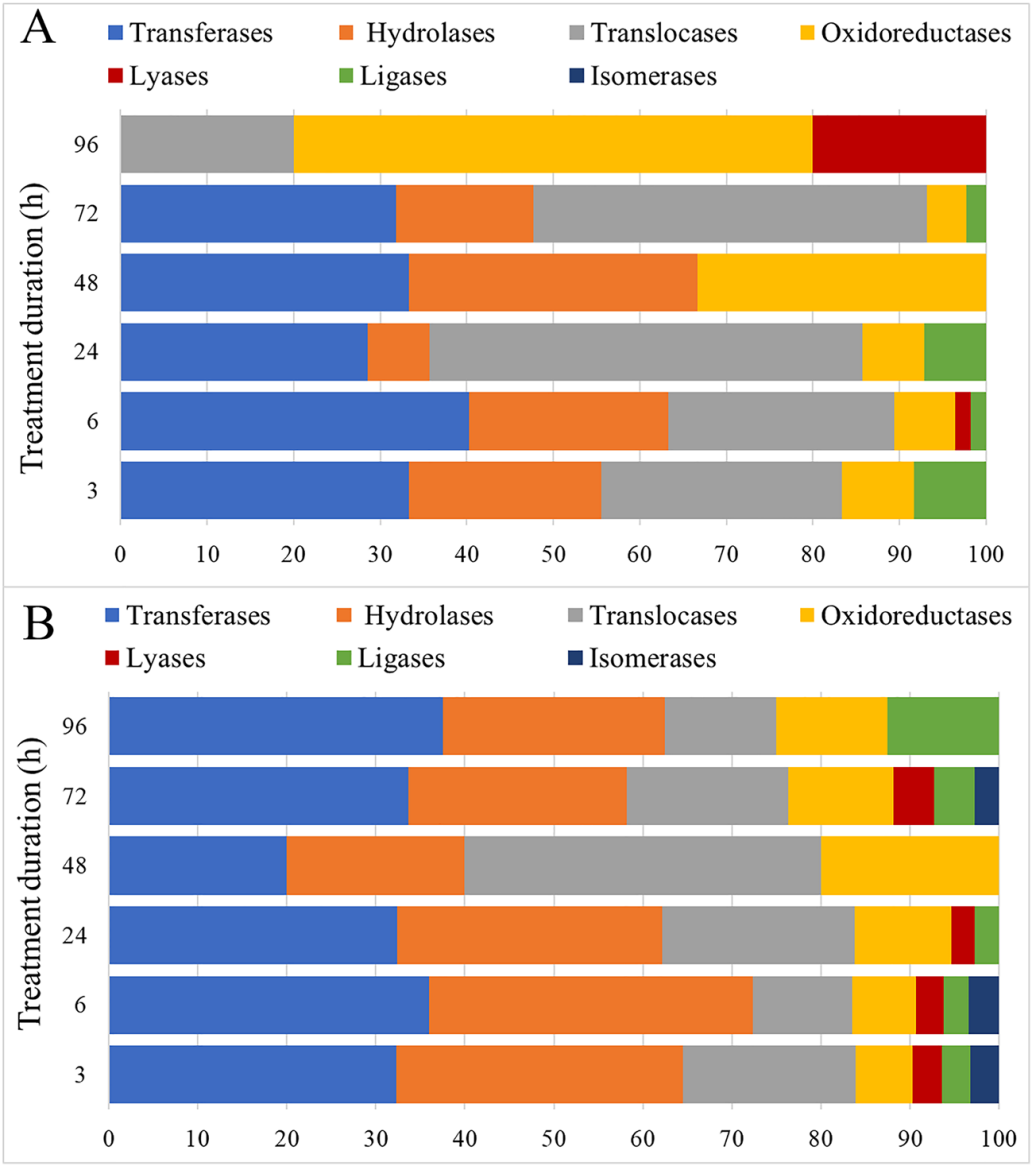
Enzyme classes represented by the (**A**) up- and (**B**) down-regulated genes at different treatment durations of *S. hasta* larva. X-axis indicates the percentage of each enzyme class and y-axis indicates the treatment duration in hours.

The significantly differentially expressed genes with a log2-fold change ≥ 1 and FDR *P* value < 0.01 at each treatment time point were considered for functional analysis using Blast2GO. The results revealed that > 90% of the differentially expressed genes had a significant BLAST match in case of both upregulated (Fig. 2A) and downregulated (Fig. 2B) lists. Furthermore, the two most common matching species based on BLAST alignment were *Acanthopagrus latus* and *Sparus aurata.* Approximately 50% of the *S. hasta* genes matched to *A. latus*. In addition, approximately 65% of the significantly differentially expressed genes had Gene Ontology annotations, while > 35% of the *S. hasta* genes had KEGG pathway annotations across various treatments. It is noteworthy that a higher fraction of the genes regulated at early and late treatments encoded for enzymes than those regulated during intermediate treatment. Furthermore, 50% of the enzymes encoded by the up/down-regulated genes belonged to transferase or translocase classes, whereas < 10% of the enzymes belonged to lyases, ligases and isomerases together (Fig. 3). Additionally, a higher percentage of the hydrolases (28%) were encoded by the downregulated genes than that of the upregulated genes (17%).

### Pathways regulated by differentially expressed genes in response to KCO-WAF treatment at different durations

The pathway analysis of the up-and down-regulated genes following KCO-WAF treatment at different durations resulted in a varying number of pathways at each time point (Table 3). Highest number of pathways were represented by the genes differentially regulated at 6 h WAF treatment, and the least number of pathways by the genes regulated at 96 h WAF treatment. Table 4 lists top 10 differentially expressed genes between the treated and untreated samples at each time point along with their annotation details and number of associated pathways. Interestingly, even though a comparatively lower number of genes were differentially expressed at 24 h WAF treatment than that at 3 h treatment, a higher number of pathways were regulated at 24 h indicating the modulation of different pathways by KCO-WAF at 24 h post exposure. Supplementary file 4 lists all the pathways represented by the differentially expressed genes at each time point.

**Table 3:** Number of pathways represented by the up and down-regulated genes following KCO-WAF treatment.

Duration of the KCO-WAF exposure (h)	No. of pathways		No. of genes annotated in pathways	
	Up	Down	Up	Down
3	173	66	48	30
6	297	334	244	293
24	184	147	31	49
48	85	140	28	42
72	214	220	72	118
96	79	44	17	12

**Table 4:** Top 10 differential genes in KCO-WAF treated compared to untreated samples at different treatment time points.

*S. hasta* gene ID	Log2 fold	FDR *P* value	BLAST match gene	No. of KEGG pathways
3 h KCO-WAF treatment				
jg9031	14.57	0.0043	No match	None
jg38000	5.62	0.0015	No match	None
jg38007	4.31	0.0015	Cytochrome P450 1B1	13
jg23177	4.24	0.0015	No match	None
jg15731	3.40	0.0015	Forkhead box protein F2a	None
jg3601	− 2.74	0.0015	Receptor-type tyrosine-protein phosphatase F isoform X14	4
jg10273	− 2.64	0.0015	Meiosis regulator and mRNA stability factor 1 isoform X4	None
jg12463	− 2.59	0.0015	DNA (cytosine-5)-methyltransferase 3A isoform X4	1
jg12242	− 2.56	0.0043	Rho guanine nucleotide exchange factor 2	None
jg27203	− 2.40	0.0015	DNA-directed RNA polymerase II subunit RPB1	None
6 h KCO-WAF treatment				
jg11940	6.21	0.0002	Glutamate receptor 3-like	13
jg22311	5.93	0.0085	No match	4
jg23177	5.74	0.0002	No match	None
jg40529	5.44	0.0026	CAC1A protein	None
jg28642	5.44	0.0018	Hypothetical protein F7725_003006	None
jg29168	− 5.05	0.00018	Extracellular calcium-sensing receptor-like	2
jg35914/jg35915	− 4.16	0.00018	Uncharacterized protein LOC109616948 isoform X2	None
jg35902	− 3.96	0.00018	Fibronectin type III domain-containing protein 7-like	None
jg27334	− 3.91	0.00093	Triadin-like isoform X1	None
jg12310	− 3.85	0.00018	Cartilage intermediate layer protein 1-like	None
24 h KCO-WAF treatment				
jg28323	6.92	0.0006	Zinc finger SWIM domain-containing protein 7	None
jg28645	2.93	0.0006	No match	None
jg30650	2.72	0.0006	No match	None
jg36960	2.66	0.0006	Receptor-type tyrosine-protein phosphatase U-like	None
jg40073	2.64	0.0006	No match	None
jg27865	− 2.98	0.0006	Hypothetical protein F7725_010018	None
jg6632	− 2.50	0.0016	FERM and PDZ domain-containing protein 2	None
jg2859	− 2.38	0.0006	2-oxoglutarate dehydrogenase-like, mitochondrial	3
jg12891	− 2.26	0.0011	Double C2-like domain-containing protein beta	None
jg39091	− 2.26	0.0090	Inositol 1,4,5-trisphosphate receptor type 1 isoform X8	53
48 h KCO-WAF treatment				
jg23775	4.80	0.0006	Hypothetical protein cypCar_00048485	3
jg41026	3.37	0.0006	Hypothetical protein FQN60_001764	None
jg20977	3.05	0.0006	KN motif and ankyrin repeat domain-containing protein 4 isoform X1	None
jg1865	2.89	0.0006	Hypothetical protein E3U43_008728	None
jg31125	4.80	0.0006	Dual specificity testis-specific protein kinase 2	None
jg1077	− 53.07	0.0006	eosinophil peroxidase-like	6
jg22257	− 47.57	0.0006	beta-crystallin B1-like	None
jg22256	− 45.24	0.0006	crystallin, beta A1, like 1	None
jg34203	− 35.11	0.0006	gamma-crystallin M2-like	None
jg34204	− 28.73	0.0006	gamma-crystallin M2-like	None
72 h KCO-WAF treatment				
jg35482	5.37	0.00031	No match	None
jg14686	3.09	0.00031	No match	None
jg28642	3.08	0.00031	Hypothetical protein F7725_003006	None
jg40872	2.74	0.00031	Uncharacterized protein LOC119027017	None
jg33295	2.71	0.00031	E3 ubiquitin-protein ligase NEURL3	None
jg9031	− 11.90	0.00031	No match	None
jg34114/jg34115/jg34116	− 5.16	0.00031	Fish-egg lectin-like	None
jg33390	− 3.61	0.001787	Uncharacterized threonine-rich GPI-anchored glycoprotein PJ4664.02-like isoform X7	None
jg5392	− 3.42	0.003073	Calymmin isoform X1	None
jg24368	− 3.38	0.00031	Melanotransferrin	None
96 h KCO-WAF treatment				
jg9031	9.51	0.003276	No match	None
jg4278	4.17	0.003276	Complexin-4	1
jg13468/jg13469	3.01	0.003276	Cingulin-like protein 1 isoform X1	1
jg8669/jg8670	2.50	0.003276	MAGUK p55 subfamily member 4 isoform X1	None
jg13267	2.17	0.003276	Protein mono-ADP-ribosyltransferase PARP6-like	None
jg34114/jg34115/jg34116	− 3.29	0.003276	Fish-egg lectin-like	None
jg1077	− 2.80	0.003276	Eosinophil peroxidase-like	6
jg12540	− 2.50	0.003276	Urokinase plasminogen activator surface receptor-like	None
jg38921	− 1.97	0.003276	CD83 antigen	None
jg10643	− 1.89	0.003276	Olfactomedin-like	None

The genes are sorted based on decreasing fold change. Up and down-regulated genes are indicated with positive and negative fold changes, respectively. The “*S. hasta* gene ID” corresponds to the gene IDs assigned by the BRAKER2 gene prediction pipeline. Details of the KEGG pathways can be found in Supplementary file 4.

**Figure 4:**
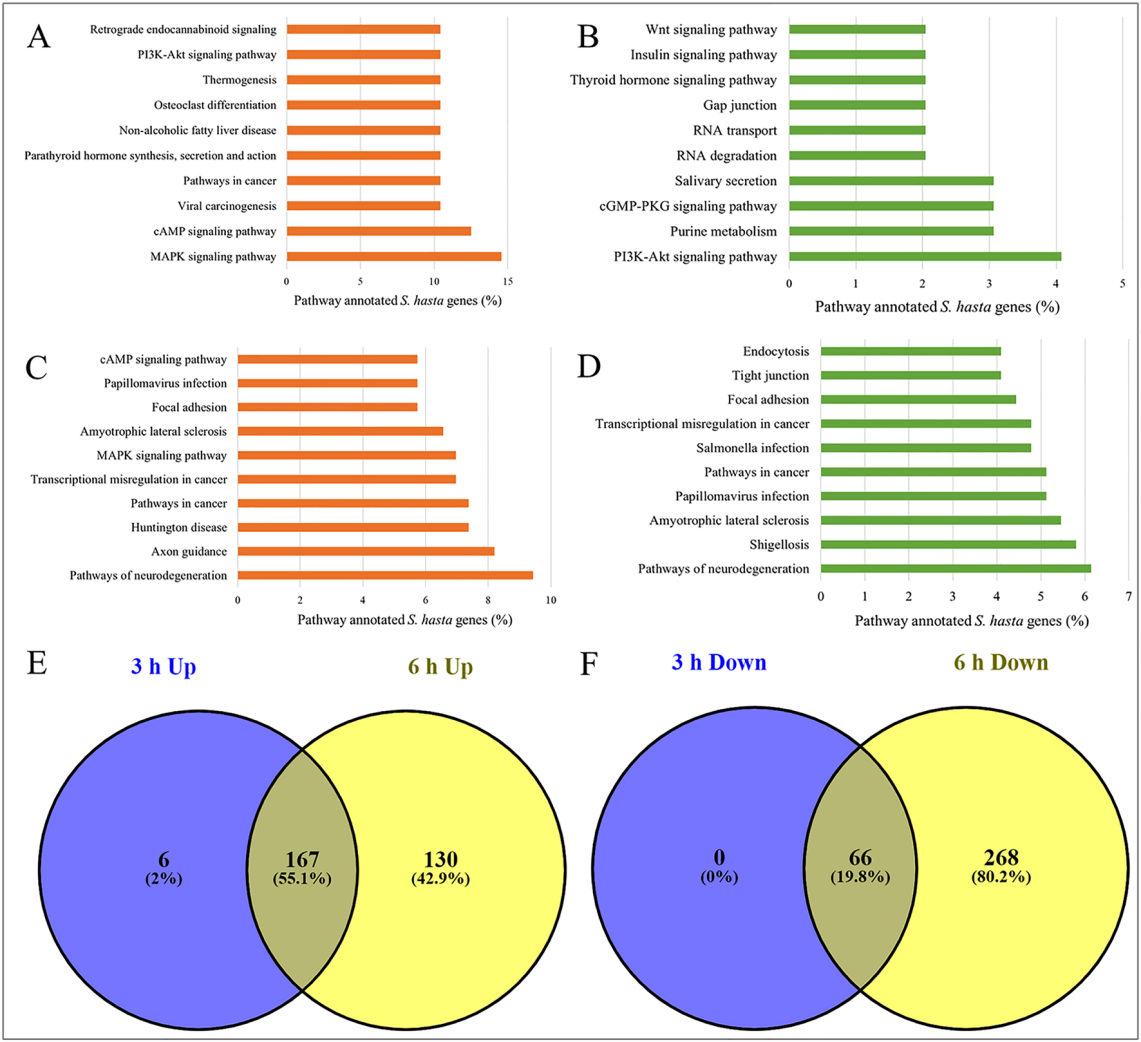
Pathways represented by the early-responsive genes following KCO-WAF treatment of *S. hasta* larva. Top 10 pathways represented by the genes up and downregulated at 3 h (**A**, **B**, respectively) or 6 h (**C**, **D**, respectively) WAF exposure. Pathways shared by early responsive up- (**E**) or downregulated (**F**) genes.

**Figure 5:**
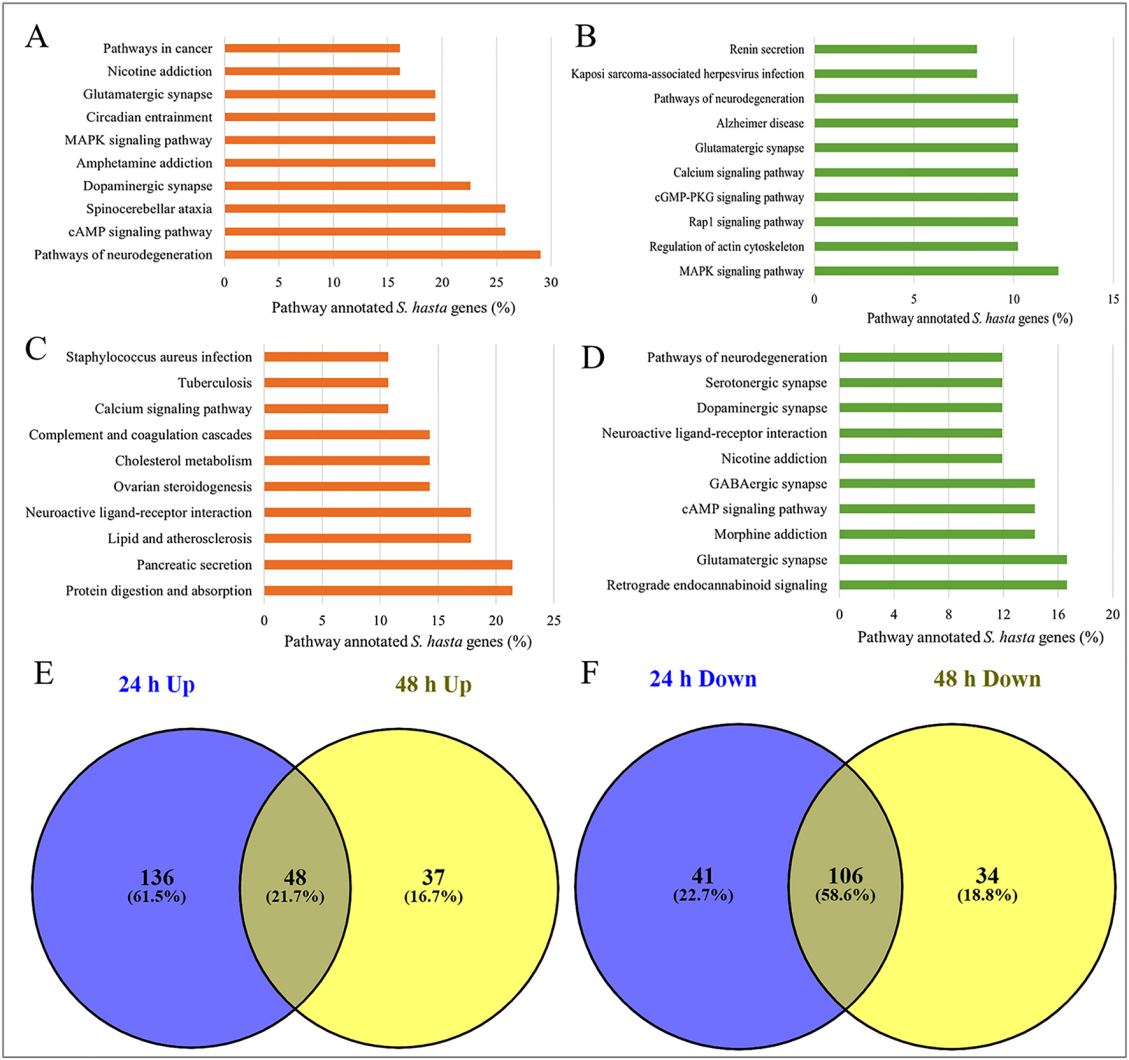
Pathways represented by the intermediate-responsive genes following KCO-WAF treatment of *S. hasta* larva. Top 10 pathways represented by the genes up and downregulated at 24 h (**A**, **B**, respectively) or 48 h (**C**, **D**, respectively) WAF exposure. Pathways shared by intermediate-responsive up- (**E**) or downregulated (**F**) genes.

**Figure 6:**
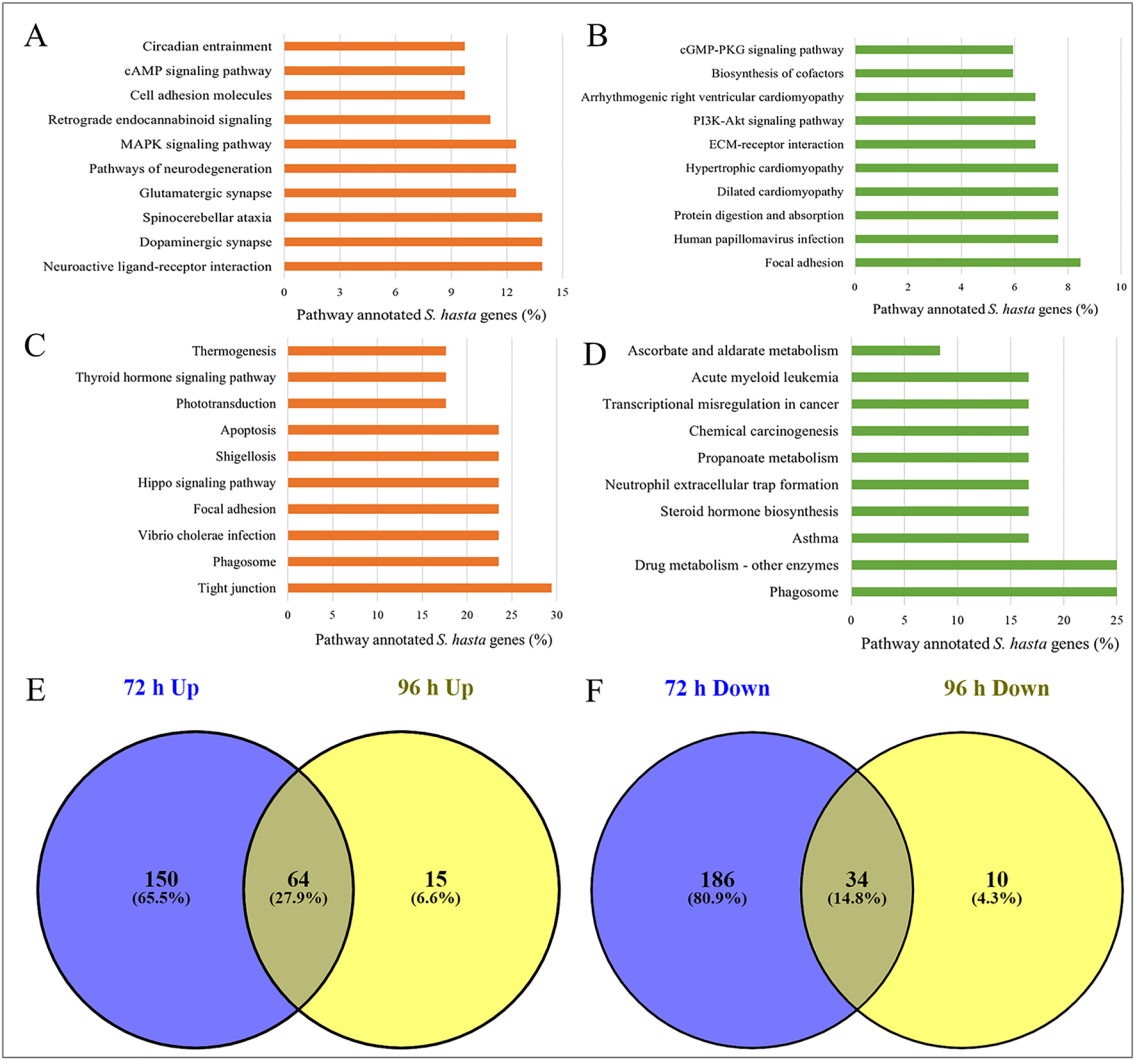
Pathways represented by the late-responsive genes following KCO-WAF treatment of S. *hasta* larva. Top 10 pathways represented by the genes up and downregulated at 72 h (**A**, **B**, respectively) or 96 h (**C**, **D**, respectively) WAF exposure. Pathways shared by late-responsive up- (**E**) or downregulated (**F**) genes.

Furthermore, the genes differentially expressed at 3 h WAF treatment were found to be involved in various signaling pathways (Fig. 4A,B, and Supplementary file 4). Among these, ‘MAPK signaling’ pathway was represented by highest number of upregulated genes (~ 15%), whereas, only 1% of the downregulated genes were involved in this pathway. Interestingly, in addition to various signaling pathways, neurological physiology related pathways were represented by the genes differentially expressed at 6 h (Fig. 4C,D) and 24 h (Fig. 5A,B) post exposure to WAF. These include ‘pathways of neurodegeneration’, ‘axon guidance’, ‘glutamatergic synapse’, ‘Alzheimer’s disease’, ‘GABAergic synapse’, and ‘serotonergic synapse’. The ‘pathways of neurodegeneration’ was represented by the highest number of upregulated genes by both 6 and 24 h post exposure to WAF. The pathways ‘protein digestion and absorption’ and ‘pancreatic secretion’ were represented by highest number of upregulated genes (~ 21%), whereas, ‘retrograde endocannabinoid signaling’ and ‘glutamatergic synapse’ were represented by most number of downregulated genes (~ 17%) at 48 h post exposure to WAF (Fig. 5C,D). Furthermore, 72 h post exposure to WAF showed upregulated pathways, related to ‘neuroactive ligand-receptor interaction’, ‘dopaminergic synapse’, and ‘spinocerebellar ataxia’, while downregulated pathways, such as ‘focal adhesion’, and ‘protein digestion and absorption’ (Fig. 6A,B). The ‘tight junction’ pathway was represented by highest number of upregulated genes (~ 29%), and ‘phagosome’ and ‘drug metabolism’ pathways were represented by most number of downregulated genes (25%) at 96 h WAF treatment (Fig. 6C,D).

### Common and exclusive pathways regulated by early-, intermediate-, and late-responsive genes following KCO-WAF treatment

Furthermore, we identified pathways that were common and exclusive to early, intermediate, or late responsive genes. The intermediate responsive genes (differentially regulated at 24 and 48 h post exposure to WAF) had highest percentage of shared pathways with 21.7% upregulated and 58.6% downregulated (Fig. 5E,F) followed by early responsive genes (differentially regulated at 3 and 6 h post exposure to WAF) with 55.1% upregulated and 19.8% downregulated pathways (Fig. 4E,F). The late responsive genes (differentially regulated at 72 and 96 h WAF treatment) shared 27.9% upregulated and 14.8% downregulated (Fig. 6E,F) pathways. Supplementary file 5 provides a list of common and exclusive pathways regulated by early-, intermediate-, and late-responsive genes expressed in response to exposure to KCO-WAF.

The genes upregulated at 3 and 6 h WAF treatment regulated various signaling pathways. A few of these pathways include, MAPK, cAMP, ErbB, TNF, PI3K-Akt, oxytocin, and glucagon signaling. The pathways exclusively represented by the upregulated genes at 3 h treatment include ‘glycolysis/gluconeogenesis’, ‘bisphenol degradation’ and that represented by upregulated genes at 6 h treatment include ‘proteoglycans in cancer’, ‘lysine degradation’, ‘insulin signaling pathway’. Similarly, the genes downregulated at 3 and 6 h treatment represented various signaling pathways. Genes downregulated at 6 h WAF treatment represented 268 exclusive pathways (Fig. 4F), including ‘Hippo signaling pathway’, ‘regulation of actin cytoskeleton’, ‘protein digestion and absorption’, ‘fatty acid degradation’, and ‘endocytosis’ (Supplementary file 5).

A total of 48 pathways were commonly upregulated at 24 and 48 h post exposure to WAF, mostly including signaling related pathways. Among these, ‘cAMP signaling pathway’ was represented by 26% of the pathway annotated upregulated genes at 24 h treatment, whereas ‘pancreatic secretion’ pathway was represented by most number (21%) of pathway annotated upregulated genes at 48 h treatment. Furthermore, the genes upregulated at 24 h treatment represented 136 exclusive pathways, whereas 37 pathways were exclusive to the upregulated genes at 48 h treatment (Fig. 5E). Similarly, 41 pathways were exclusive to genes downregulated at 24 h WAF treatment, whereas 34 pathways were exclusive to the genes downregulated at 48 h treatment (Fig. 5F). Among the exclusive pathways, ‘pathways of neurodegeneration’ was represented by 29% of annotated genes upregulated at 24 h treatment, whereas ~ 21% of the annotated genes were involved in ‘protein digestion and absorption’ among those upregulated at 48 h treatment. When downregulated genes were considered, 106 pathways were commonly regulated by 24 and 48 h WAF treatment, whereas 41 and 34 pathways were exclusively regulated by 24 and 48 h treatment, respectively. ‘Axon guidance’ was exclusively regulated by 8.2% of the pathway annotated genes that were downregulated at 24 h, whereas, each of the ‘retinol metabolism’ and ‘Hippo signaling’ pathways were regulated by 4.8% of the pathway annotated genes that were downregulated at 48 h treatment (Supplementary file 5).

Furthermore, a total of 64 pathways were commonly upregulated by late WAF treatment (72 and 96 h treatment). Among these, ‘neuroactive ligand-receptor interaction’ was represented by 14% of the pathway annotated genes upregulated at 72 h treatment, whereas ‘tight junction’ pathway was represented by most number (29.4%) of pathway annotated genes upregulated at 96 h treatment. In addition, the genes upregulated at 72 h treatment represented 150 exclusive pathways, whereas 15 pathways were exclusive to the upregulated genes at 96 h treatment (Fig. 6E). ‘Dopaminergic synapse’ and ‘spinocerebellar ataxia’ pathways were each exclusively represented by 14% of the pathway annotated genes that were upregulated at 72 h treatment, whereas, ‘phagosome’ was exclusively represented by 23.5% of the pathway annotated genes that were upregulated at 96 h treatment. There were 34 pathways that were commonly regulated by the genes downregulated at 72 and 96 h treatment, while 186 and 10 pathways were exclusive to the downregulated genes at 72 h and 96 h treatments, respectively (Fig. 6F).

### RT-qPCR validation of differentially expressed genes

RT-qPCR was performed to validate the RNA sequencing results. The validation was performed by randomly selecting 12 genes among those that were significantly differentially expressed (FDR *P* value < 0.05) in at least one treatment group based on RNA sequencing. The differential regulation obtained by the RT-qPCR analysis showed complete agreement with that of the RNA sequencing data for the validated genes (Table 5).

**Table 5:** RT-qPCR validation of differentially expressed transcripts following KCO-WAF exposure.

Transcript identifier		BLAST matched SwissProt name (BLAST2GO percentage identity)	Fold Change	
			RNA-Seq	RT-PCR
3 h post exposure				
jg9031.t1	Up	Synaptosomal-associated protein 25 (98.28)	14.57	3.63
jg38000.t1	Up	–	5.62	1.19
jg38007.t1	Up	Cytochrome P450 1A1 (62.4)	4.31	5.64
jg35482.t1	Up	–	0.90	1.29
jg6070.t1	Down	–	− 2.04	− 1.84
jg7604.t1	Down	Tether containing UBX domain for GLUT4 (80.13)	− 2.17	− 2.13
6 h post exposure				
jg38000.t1	Up	–	2.37	0.59
jg38007.t1	Up	Cytochrome P450 1A1 (62.4)	4.38	3.50
jg6070.t1	Down	–	− 2.22	− 1.03
jg7604.t1	Down	Tether containing UBX domain for GLUT4 (80.13)	− 2.41	− 0.39
jg19122.t1	Down	Tetraspanin-1 (53.73)	− 3.13	− 5.09
24 h post exposure				
jg38007.t1	Up	Cytochrome P450 1A1 (62.4)	1.42	4.94
jg35482.t1	Up	–	2.24	0.76
jg7604.t1	Down	Tether containing UBX domain for GLUT4 (80.13)	− 0.59	− 1.20
jg38623.t1	Down	Protein transport protein sec1 (63.28)	− 1.58	− 0.35
jg21630.t1	Down	WW domain-containing oxidoreductase (56.26)	− 0.46	− 5.04
48 h post exposure				
jg38000.t1	Up	–	0.69	1.28
jg38007.t1	Up	Cytochrome P450 1A1 (62.4)	1.35	1.22
jg4278.t1	Down	Complexin-4 (72.04)	− 2.05	− 0.74
jg38623.t1	Down	Protein transport protein sec1 (63.28)	− 1.37	− 0.01
jg21630.t1	Down	WW domain-containing oxidoreductase (56.26)	− 1.28	− 0.47
72 h post exposure				
jg9031.t1	Down	Synaptosomal-associated protein 25 (95.58)	− 11.90	− 3.19
jg38000.t1	Down	–	− 0.62	− 4.90
jg6070.t1	Down	–	− 0.64	− 2.97
jg4278.t1	Down	Complexin-4 (72.04)	− 1.76	− 5.20
96 h post exposure				
jg3323.t1	Down	Lumican (63.97)	− 1.10	− 1.88
jg36772.t1	Down	Protein eva-1 homolog C (53.75)	− 1.76	− 0.78

## Discussion

Anthropogenic activities, such as crude oil contamination, have a significant impact on the marine ecosystem and the health of fish. The release of crude oil into the marine environment can occur through natural disasters, such as oil spills, or through human activities, such as oil exploration and transportation. The impacts of crude oil contamination on the marine ecosystem and fish health can be devastating, and the effects can be long-lasting. It is crucial to investigate the impact of oil pollution on fish species. While, oil pollutants greatly affect the adult fish, their deleterious effects are much more pronounced in the larval stages. Consequently, several studies have focused on examining the damage caused to fish larvae or eggs when exposed to crude oil^9,18,19^. Here we further explore the impacts of crude oil contamination on the marine ecosystem and fish health, with a focus on the changes in the gene expression which results in alteration in the regulation of genetic pathways during the early developmental stages of fish larvae.

In the current study, we exposed *S. hasta* larvae to KCO-WAF for varying durations (3, 6, 24, 48, 72, or 96 h) and explored its deleterious effects on the gene expression using RNA sequencing. Additionally, we studied the functional processes and pathways that are modulated at different exposure times.

The use of WAF of crude oil is a well-established method to test the toxicity of petroleum^31,41,42^. However, it has some limitations mainly due to minor differences in the technique used for WAF preparation which can alter the composition of the WAF in different laboratories. Also, the chemical composition of the crude oil can vary depending on the source and batch, and thus the water WAF composition could vary. The selection of KCO-WAF concentrations in our study was based on established practices in the field of aquatic toxicity testing for petroleum products^24,28,43–48^. Use of WAF has emerged as the method of choice for its simplicity and wide applicability over three decades, allowing for the testing of complex substances like petroleum products in biological systems^48^. In the aquatic toxicity tests, the exposure concentration primarily represents the bioavailable dissolved fraction of the test material. WAF based toxicology experiments has proven effective across various organisms, including algae, crustaceans, gastropod and bivalve mollusks, and fish, both in freshwater and saltwater environments^48^. The utilization of WAF concentrations in our study aligns with the well-established and widely adopted methodology in the field of aquatic toxicity assessment for petroleum products.

Earlier reports demonstrated the degree of toxicity of WAF prepared from varying KCO loadings conducted in exposure chambers with fish larvae exposed for 96 h^28,49^. In this study, the total petroleum hydrocarbon (TPH) concentrations in the KCO-WAF prepared using 0.5 g /L oil loading in seawater was found to be 0.4 ± 0.09 mg/L. Although the WAF exposure experiments with sobaity larvae were conducted at three different KCO oil loading (0.25, 0.5, 1 g/L), the results indicated that 0.5 g/L KCO loading for WAF preparation is optimum for crude oil exposure assays (data not shown). Earlier studies also have highlighted that the acute toxicity in crude oils is primarily attributed to the presence of polycyclic aromatic hydrocarbons (PAHs), with naphthalene being a significant contributor^50^.

Environmental changes due to anthropogenic activities can have detrimental affect on the aquatic organisms. Scott and Sloman discussed how various toxicants disrupt complex fish behaviors essential for fitness and survival, often at lower exposures than those causing mortality^20^. In this context, crude oil pollution of aquatic habitat has been shown to exhibit profound negative effects on fish species especially at the early larval stages. Incardona et al. found that exposure to crude oil-derived PAHs caused specific dose-dependent defects in cardiac function in pelagic fish species, indicating potential mortality and malformations^51^.

Understanding the early changes in gene expression in fish larvae exposed to crude oil toxicity represents a crucial area of research with several notable gaps. While there is a growing body of literature examining the impacts of crude oil exposure on fish development, there is a paucity of comprehensive studies focused specifically on the gene expression changes in the early developmental stages of larvae. Understanding the molecular responses during this critical period is essential for elucidating the mechanisms underlying developmental abnormalities and impaired growth observed in fish exposed to crude oil. Additionally, there is a need for more extensive investigations into the specific genes and pathways that are modulated early on, as this information could provide valuable insights into the biomarkers indicative of oil-induced stress and potential targets for mitigation strategies.

Studying the immediate effects of crude oil on fish larvae, ranging from 3 to 96 h of acute exposure, allows us to capture the early molecular responses triggered by crude oil exposure, providing insights into the immediate changes in gene expression and signaling pathways. The acute exposure period is critical for understanding the initial impact of crude oil on biological systems, as it helps identify the immediate stress responses and potential adaptive mechanisms activated by the organisms. The differential expression analysis of genes indicated that WAF treatment at 6 h had a profound effect on the fish larvae showing higher number of differentially expressed genes. These findings were further corroborated by pathway analysis, which showed modulation of most pathways by the genes deregulated at 6 h treatment. Most of the top 10 genes were annotated to known genes using BLAST, however, very few of these had pathway annotations, which indicates that the differencially expressed genes in response to KCO-WAF need to be further investigated in terms of their role. When we grouped the treatments into early (3 and 6 h), intermediate (24 and 48 h) and late (72 and 96 h) exposure, the genes modulated by the intermediate exposure were found to be similar in function based on the pathways they shared, whereas, a higher percentage of pathways were unique during the late exposure. Thus, different sets of pathways are regulated by KCO-WAF exposure in a time-dependent manner.

The activation of multiple genes associated with crucial cellular signaling pathways (Fig. 4), such as MAPK (Mitogen-Activated Protein Kinase), cAMP (cyclic adenosine monophosphate), and PI3K-Akt (Phosphoinositide 3-kinase-Akt), within a short period of 3–6 h post crude oil exposure suggests a rapid and dynamic cellular response to environmental stress. These signaling pathways play pivotal roles in various cellular processes, including cell proliferation, survival, and response to external stimuli. Crude oil exposure can lead to oxidative stress and dysfunction in various organisms. Earlier reports suggests uprgulation of CYP450 in response to crude oil exposure^43,46,47,52–54^. Cytochrome P450 is a xenobiotic-metabolizing enzyme, and its upregulation suggests a detoxification response to the presence of crude oil contaminants. Members of the CYP450 family, belonging to 1A and 1B groups are induced by aryl hydrocarbon receptors (AhRs) upon binding of PAH ligands and results in enhanced metabolism of xenobiotics^55^. Rapid upregulation of Cytochrome P450 1B1 gene was detected in our study at 3 h of post exposure to WAF (Table 4). The effectiveness of the detoxification response in mitigating the toxic effects of crude oil is still a subject of research. In earlier studies it has been shown that exposure to elevated crude oil concentrations induces a lethal syndrome of heart failure in fish embryos, primarily attributed to cardiotoxic polycyclic aromatic hydrocarbons (PAHs) found in petroleum. Even low concentrations of oil during embryonic development result in sublethal toxicity, which persists and is not mitigated by Cytochrome P450 induction^56^. The findings could have profound implications beyond fish populations, indicating potential impact of such pollution for other vertibrates including human^56^.

The MAPK pathway is a well-known signaling cascade that regulates cellular responses to a variety of stimuli, including environmental toxins^57^. The rapid activation of MAPK genes implies that fish larvae are mobilizing intracellular signaling mechanisms to adapt to the stress induced by crude oil exposure^58–60^. This early response could be indicative of the early protective mechanisms or repair processes. The upregulation of genes associated with cAMP suggests that fish larvae are orchestrating a complex intracellular communication network in response to crude oil exposure. This could involve modulation of various cellular processes, including gene expression, metabolism, and stress responses as cAMP is a key messenger that mediates cellular responses to extracellular signals^61–64^. The involvement of the PI3K-Akt pathway further highlights the complexity of the cellular response. This pathway is central to regulating cell survival and growth, and its activation may indicate an effort to maintain cellular homeostasis under the stress of crude oil exposure^65–69^. The upregulation of genes such as synaptosomal-associated protein and cytochrome P450 1B1 indicates its possible role in synaptic vesicle trafficking and neurotransmitter release, suggesting that crude oil exposure may influence neural processes in fish larvae.

In summary, the changes in gene expression indicates an orchestrated molecular response in sobaity fish larvae to crude oil exposure. Understanding these early changes at the genetic level provides valuable insights into the mechanisms of adaptation and potential stress-induced impacts on various cellular processes. Further research into the functional consequences of these gene activations can help unravel the intricacies of the biological response to environmental stressors.

Furthermore, our functional analysis showed that many of the differentially expressed genes matched to transferase or translocase enzyme classes. Transferases are the class of enzymes that catalyze the transfer of specific functional groups from one molecule to another^70^. These classes of enzymes are involved in various biochemical pathways and integral to most of the important biological processes, e.g., translation^71^. Furthermore, translocases are class of enzymes that catalyze the movement of ions or molecules across membranes^72^. These classes of enzymes are involved in process, such as oxidative phosphorylation, electron transport chain, and fatty acid oxidation^70–74^. Thus, a higher percentage of the genes coding for such enzymes being differential clearly indicates the modulation of various metabolic pathways in fish larvae following WAF exposure.

The differentially expressed genes modulated various pathways, including those related to signaling, neurodegeneration, nervous system, and different diseases. These pathways are fundamental to the intricate network of molecular interactions that regulate critical aspects of an organism's development, physiological functions, adaptations and stress tolerance. By modulating these pathways, cells can respond to various internal and external cues, ensuring the proper progression of developmental stages and the coordination of essential physiological activities during growth and development. Understanding the intricacies of these pathways is important to study the molecular mechanisms that underline stress tolerance in *S. hasta* larvae exposed to chemical pollutants including WAF of crude oil.

Disruption of calcium signaling pathways has been known to result in cardiac morphology abnormalities^75,76^, and leads to defects in cardiac rhythm and contractility, as well as cardiomyocyte proliferation^51,77^. Our results revealed that differentially expressed genes at the 24-h and 48-h treatment time points were associated with the 'calcium signaling pathway,' suggesting that WAF exposure impacts cardiac functions in *S. hasta* fish larvae (Fig. 5). At 24 h post-exposure, a higher percentage of genes associated with 'calcium signaling pathway,' were downregulated, whereas at 48 h, a higher percentage of genes were found to be up regulated (Fig. 5). Furthermore, we found a higher number of genes to be associated with neurodegenerative disorders and other neurology-related pathways when fish larvae were exposed to WAF for longer duration. Thus, the crude oil exposure for extended duration may result in neurological damages in developing fish larvae. Our findings are in agreement with the previous whole transcriptome studies, which demonstrated that exposure to crude oil components result in physiological changes in the brain tissue, neurotransmitter regulation, and locomotory behaviour in fish species, indicating a neurotoxic potential of the petroleum constituents^78–80^. Furthermore, Xu et al. demonstrated time-dependent transcriptomic and physiological changes in embryos/larvae of mahi-mahi exposed to WAF of weathered (slick) oil^47^. The predominant transcriptomic responses upon slick oil exposure included alteration of signaling, ribosome biogenesis, steroid biosynthesis, and activation of the Cytochrome P450 pathway, corroborating with the findings from the current study. Additionally, 96 h of exposure to WAF resulted in significant perturbations in the eye and peripheral nervous system which was in agreement with our findings. A transcriptomic study on mahi-mahi indicated a switch in the developmental progression based on the enrichment of different biological processes and pathways while progressing from early embryonic stage to late developmental stages^81^.

Earlier studies predominantly focused on juvenile or mature fishes, leaving a critical gap in understanding how gene expression changes over time in the early life stages of fish. This lack of knowledge is particularly significant as fish larvae represent a vulnerable stage, and their responses to environmental stressors may differ substantially from those of adults. An ecotoxicogenomic study on juvenile rainbow trout exposed to crude oil, temporal changes in gene expression were examined, revealing unique patterns in gill and liver responses^82^. While the gill exhibited significant alterations in the expression of several genes during exposure, the liver showed delayed responses, with concentration-dependent effects^82^. A high number of 1137 genes were differentially regulated within 24 h of exposure in the gill tissues. The observed rapid response of larvae at early developmental stages aligns with findings in the study on juvenile rainbow trout exposed to crude oil, where a high number of pathways/genes were differentially regulated during initial exposure at 24 h. There number of pathways gradually decreased at later stages (72 and 96 h post-exposure) in *S. hasta* larvae. The late-responsive genes identified in our study were particularly those associated with the nervous system, phagosome, and dopaminergic synapse, parallel the neurodegenerative/nervous system-related pathways. Prolonged 96 h exposure to KCO-WAF resulted in the manifestation of symptoms such as abnormal and slow swimming, musculoskeletal deterioration leading to impaired mobility and body control which typically mirrors the deleterious effects of crude oil exposure as shown in previous work by others^28,32,33,49,83^. Our study also showed an enrichment of different sets of pathways in *S. hasta* larvae following WAF treatment in a time-dependent manner, showing that WAF exposure for longer durations have significant deleterious effects on fish larvae.

The current study used RNA sequencing to reveal the effects of KCO-WAF on the developmental phases of *S. hasta*. Different sets of genes were found to be differentially expressed following WAF exposure at 3, 6, 24, 48, 72, or 96 h. WAF exposure at 6 h had the most significant effect on fish larvae, as evident from the number of differentially expressed genes. Functional analysis of the differentially expressed genes clearly indicated modulation of various physiological and developmental processes in fish larvae following WAF exposure. The pathways that were found to regulated by the differentially expressed genes at different time points included primarily signaling, cardiac, and nervous system related pathways. Thus, our results indicate that WAF exposure has deleterious effects on cardiac physiology and nervous system during the developmental stages of *S. hasta*.

### Deposited data and information to the user

The complete sequences, which were used for the genome assemblies and annotations, have been deposited in public data repositories. The DNA libraries used in draft genome assembly for *S. hasta* have been deposited in the NCBI sequence read archive (SRA: SRR17438565) under the Project ID: PRJNA794279. The RNA sequence data is available under the BioProject accession: PRJNA748027.

### Supplementary Information

Supplementary Information 1.

Supplementary Information 2.

Supplementary Information 3.

Supplementary Information 4.

Supplementary Information 5.
